# Fecal Microbiomes in Ex Situ-Managed Mammals: Captivity-Associated Shifts and Links to Welfare, Stress, and Reproductive Performance

**DOI:** 10.3390/ani16162480

**Published:** 2026-08-10

**Authors:** Mi-Rim Kwon, Dong-Hyuk Jeong

**Affiliations:** 1 Laboratory of Wildlife and Conservation Medicine, College of Veterinary Medicine, Chungbuk National University, Cheongju 28644, Republic of Korea; mirim7468@chungbuk.ac.kr; 2 Wildlife Center of Chungbuk, Cheongju 28116, Republic of Korea

**Keywords:** animal welfare, captive breeding, conservation microbiome, ex situ conservation, fecal microbiome, reproductive performance, stereotypic behavior

## Abstract

Ex situ conservation programs maintain threatened mammal populations and support conservation breeding and reintroduction, but success also depends on physiological function, behavioral competence, welfare, and reproductive performance. This critical narrative review combined structured literature searches with a descriptive quantitative evidence map of published summary values to evaluate mammalian fecal or intestinal microbiomes in relation to wild–captive transitions, diet and management, welfare-related outcomes, and reproduction. Captivity was repeatedly associated with altered microbial community composition, but richness and major phylum-level changes showed no universal direction. Host phylogeny, diet, digestive anatomy, facility, environmental exposure, reproductive state, and analytical methods constrained comparisons. Five studies provided direct Tier 1 evidence by jointly measuring microbiomes with outcomes spanning stereotypic behavior, stress and endocrine measures, breeding season, breeding success, and multigenerational fitness within the same ex situ-managed populations. These included associations with stereotypic behavior, breeding season, hormone metabolites, breeding success, and multigenerational fitness trajectories. However, the evidence remains observational and taxon-specific, and no bacterial feature is currently validated as a transferable biomarker. Longitudinal fecal microbiome monitoring may add value when integrated with behavioral, endocrine, clinical, dietary, and breeding data. Standardized repeated-measures studies, functional profiling, and prospective management interventions are priorities for translating microbiome research into evidence-based ex situ conservation.

## Simple Summary

Zoos and conservation breeding centers protect threatened mammals, but animals under human care may experience changes in diet, environment, behavior, health, and breeding performance. The microorganisms living in the digestive tract may respond to these changes and could provide useful information about how animals adapt to managed conditions. We reviewed studies of mammalian gut microorganisms in relation to captivity, feeding and housing conditions, stress-related measures, abnormal repetitive behavior, and reproduction. Captivity frequently changed gut microbial communities, but the direction of change differed among species and facilities. A small number of studies linked microbial variation with repetitive behavior, reproductive hormones, breeding season, breeding success, or changes across captive generations. These associations do not prove that gut microorganisms cause welfare or reproductive outcomes, and no single bacterial group can yet be used as a general indicator across species. Repeated fecal sampling may nevertheless strengthen conservation management when it is combined with dietary records, behavioral observations, hormone measurements, health examinations, and breeding histories. Standardized long-term studies are now needed to determine which microbial changes are biologically meaningful and whether management interventions can improve animal welfare, reproduction, and readiness for reintroduction.

## 1. Introduction

Ex situ programs conducted in zoos, conservation breeding centers, rescue facilities, and pre-release facilities are an essential complement to in situ conservation. They maintain genetically and demographically managed populations, provide opportunities for research and veterinary care, and can supply individuals for reinforcement or reintroduction [1,2]. These programs must simultaneously preserve population viability, animal health, behavioral competence, and reproductive capacity. This combination is difficult to achieve. Captive populations of some threatened mammals continue to experience poor reproductive performance, chronic disease, obesity or metabolic disturbance, and abnormal repetitive behaviors despite major advances in husbandry and veterinary medicine [1,2,3,4,5,6]. Such outcomes can compromise both welfare and the conservation value of the population.

Traditional explanations for these problems focus appropriately on nutrition, genetics, social compatibility, enclosure design, infectious disease, and endocrine function. A further layer of biological organization is provided by the host-associated gut microbiome, which contributes to nutrient transformation, epithelial integrity, immune regulation, colonization resistance, and metabolite production along microbiota–gut–brain and microbiota–gut–gonadal axes [7,8,9,10]. Experimental and observational studies in laboratory, domestic, and human systems have linked microbiome variation with metabolic, inflammatory, stress-related, and behavioral phenotypes [11,12,13,14,15,16,17]. Their direct relevance to wildlife, however, cannot be assumed because wild species differ profoundly in evolutionary history, digestive anatomy, natural diet, seasonality, and reproductive physiology.

Most primary data in this review come from fecal samples, which are practical proxies for gut communities in ex situ-managed mammals. We therefore use the term “fecal microbiome” when referring directly to fecal data and reserve “gut microbiome” for broader conceptual discussion.

Captivity itself can act as an ecological filter on host-associated microbial communities. Transition to human care commonly changes diet diversity and composition, exposure to soil, plants, prey and conspecifics, activity patterns, social contact, medication, climate, and contact with human-associated microorganisms. Comparative studies in bears, equids, marsupials, monotremes, rodents, carnivores, and primates have repeatedly detected differences between wild and captive populations [18,19,20,21,22,23,24,25,26,27,28,29,30,31,32,33]. A meta-analysis across vertebrates also reported widespread compositional change, although the magnitude and direction differed among host groups [34]. Captive conditions therefore do not produce a single, predictable microbial state. Rather, they alter the ecological processes that assemble the microbiome, with outcomes conditioned by the host and the details of management.

The conservation importance of host microbiomes was first articulated through the conservation microbiome and holobiont perspectives [35,36,37]. Subsequent reviews have considered the effects of captivity on animal microbiomes, the possible use of microbial management in conservation, and metagenomic approaches for threatened mammals [38,39,40]. Reproductive microbiomes of wild mammals have also received focused attention, particularly microbiota of the vagina, uterus, semen, and other reproductive sites [41,42,43]. A more specific gap remains at the interface of ex situ mammal management, the gut microbiome, animal welfare, and conservation breeding. Recent reviews have considered how gut microbiomes might improve captive animal health, inform conservation biology more broadly, or contribute to reproductive management in wildlife. However, none of these syntheses focus specifically on ex situ-managed mammals by jointly mapping gut microbiome changes across wild–captive transitions, within-captive management conditions, welfare indicators, and conservation breeding outcomes. Here, we integrate mammalian fecal microbiome data across wild–captive comparisons, within-facility management contrasts, stereotypic behavior, stress and endocrine measures, and reproductive performance to identify where gut microbiome monitoring is already informative for ex situ management and where evidence is still missing. Evidence relevant to this interface is spread across studies of wild–captive transitions, diet, abnormal behavior, stress endocrinology, seasonal reproduction, pregnancy, and multigenerational captive adaptation. The strength of inference also varies markedly: some studies simultaneously measure microbiome and conservation outcomes, whereas others provide only contextual or mechanistic support. Table 1 summarizes how the scope of this review differs from recent adjacent syntheses on captive animal microbiomes, conservation metagenomics, and reproductive microbiomes [38,39,40,41].

This review therefore has four objectives. First, we describe baseline variation in the gut microbiomes of ex situ-managed mammals and emphasize the roles of host diet, gut morphology, and phylogeny. Second, we evaluate captivity as a multilevel ecological filter, including transitions from the wild and variation among diets, facilities, and management intensities. Third, we critically assess evidence linking the gut microbiome with behavioral, stress-related, and reproductive outcomes. Fourth, we propose a practical framework for microbiome-informed monitoring in conservation breeding and reintroduction programs, while consistently distinguishing association from causation and avoiding simple equation of wild–captive differences with dysbiosis or poor health. In this review, we use the term “dysbiosis” descriptively, to refer to captivity- or management-associated alterations in gut microbiome structure that are plausibly linked to impaired health, welfare, or reproductive performance in a given host context, rather than as a universal diagnostic category.

## 2. Materials and Methods

### 2.1. Review Design and Search Strategy

This study was designed as a critical narrative review with a structured literature search and a descriptive quantitative evidence-mapping component. Its aim was to summarize eligible studies transparently in a table-based format and to visualize broad cross-study patterns, rather than to conduct a formal systematic review, scoping review, or meta-analysis. A formal systematic approach was not considered appropriate because the available literature is highly heterogeneous with respect to host species, management context, definitions of captivity, sampling structure, molecular methods, sequencing depth, taxonomic databases, and outcome measures.

The formal literature search was conducted in PubMed, and all numerical counts reported in the study-selection flow refer exclusively to the PubMed search set. Google Scholar was consulted only informally at the outset of the project to check topic coverage and identify a small number of seed articles. It was not used to construct the formal search set, was not systematically screened, and did not contribute directly to the numerical counts or the study-selection flow diagram. Searches were updated on 13 June 2026. Web of Science and Scopus were not searched; therefore, relevant studies indexed exclusively in those databases may have been missed.

The PubMed search was performed using the following exact query phrases, each entered directly as written: “captive mammal gut microbiome,” “ex situ conservation microbiome,” “wild captive gut microbiota,” “zoo animal gut microbiome,” “gut microbiome stereotypic behavior,” “gut microbiome stress welfare,” “gut microbiome breeding success,” and “conservation breeding microbiome.” These phrases were entered as simple keyword searches in PubMed without field restrictions, language limits, or date filters. Complete PubMed query phrases, search dates, and records retrieved are provided in Supplementary Table S1.

Across all PubMed queries, 1704 records were retrieved. After removal of 405 duplicates, 1299 records were screened. Of these, 1017 were excluded at the title-screening stage and 140 at the abstract-screening stage, leaving 142 full-text articles for eligibility assessment. Following full-text review, 69 articles were excluded and 73 studies were retained in the final evidence base.

**Figure 1:**
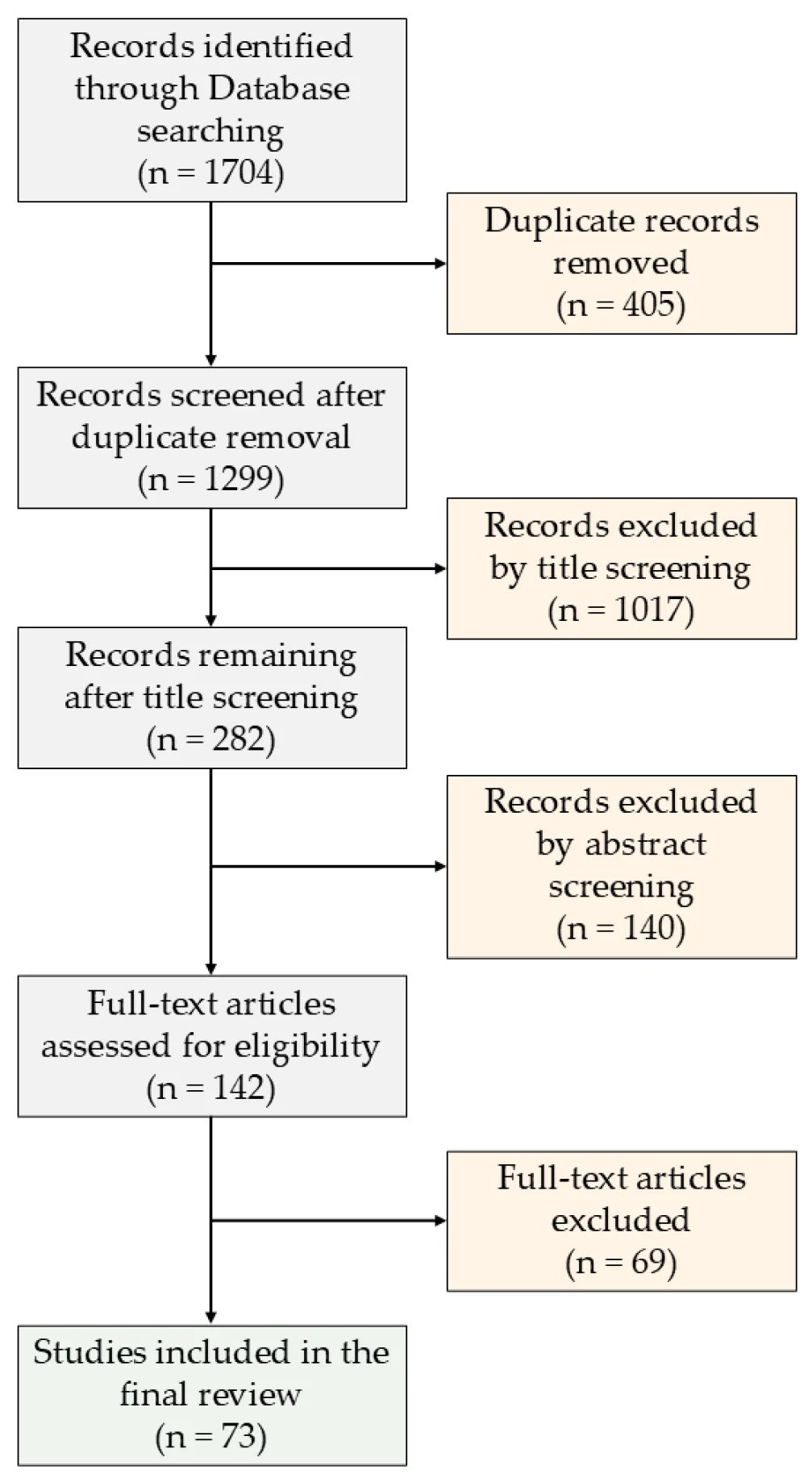
PRISMA-adapted flow diagram of study selection. Records identified, screened, and retained for this narrative evidence-mapping review of gut microbiomes in ex situ-managed mammals. The figure shows 1704 records retrieved through PubMed searches, 405 duplicates removed, 1299 titles/abstracts screened, 142 full-text articles assessed for eligibility, and 73 studies included in the final evidence base.

Because this review was narrative in design and intended as an evidence-mapping exercise, it did not include protocol registration, duplicate independent screening, pooled effect estimation, or a formal risk-of-bias appraisal. Accordingly, Figure 1 should be interpreted as a PRISMA-adapted flow summary, rather than as a fully PRISMA-compliant systematic-review diagram.

### 2.2. Eligibility and Study Selection

Studies were retained when they (i) investigated a mammalian host; (ii) included fecal, intestinal, or gut microbial data; and (iii) involved an ex situ-managed population, a wild–captive comparison, a comparison among managed conditions, or a measurable behavioral, endocrine, health, or reproductive outcome. Human, laboratory-animal, and livestock studies were excluded from the exploratory wildlife comparisons, although selected mechanistic studies were retained when they clarified plausible pathways. Studies restricted to reproductive-tract microbiota were excluded from the quantitative synthesis but were considered when defining the boundary between gut and reproductive microbiome research. Non-mammalian studies were used sparingly as mechanistic context and were not used to infer mammalian management effects. Potentially eligible reports were evaluated against these criteria, and numerical values used in the tables and figures were cross-checked against the source text, tables, figures, or supplementary files before inclusion.

### 2.3. Data Extraction and Evidence Classification

From eligible primary studies, we extracted host species, conservation or management context, wild or captive status, diet and enclosure condition, sample size where reported, sample type, microbiome method, alpha-diversity measure, major phylum-level relative abundances, and behavioral, endocrine, clinical, or reproductive outcomes. Taxonomic nomenclature was harmonized at the phylum level where possible; for example, Bacteroidetes and Actinobacteria were reported as Bacteroidota and Actinobacteriota in the synthesis, whereas other major phyla (such as Firmicutes, Proteobacteria, and Cyanobacteria) were retained under the names used in the source papers. Numerical values were extracted by two authors (M.-R.K. and D.-H.J.). For each eligible study, one author performed primary extraction from the text, tables, figures, and supplementary files, and the second author independently checked all values against the original source. Discrepancies were resolved by returning to the published article and, when necessary, by joint review at the full-text PDF or supplementary file level. Only values that could be unambiguously matched to a specific management context and group (for example, captive vs. wild, facility, diet, or reproductive category) were retained.

For the purposes of this review, the evidence linking microbiomes with welfare or reproduction was classified into three tiers. The tiers were intended to distinguish the directness of linkage between microbiome measures and conservation-relevant outcomes, rather than to grade overall methodological quality, risk of bias, or certainty of evidence. Tier 1 comprised direct ex situ evidence in which microbiome data and the relevant behavioral, endocrine, or reproductive outcome were measured in the same managed population. Tier 2 comprised contextual ex situ evidence in which microbiome patterns or conservation outcomes were measured, but not jointly, or where separate studies in the same conservation system supported a plausible association. Tier 3 comprised mechanistic support from free-ranging wildlife, domestic species, or experimental models. This classification was used to prevent mechanistic plausibility from being presented as direct evidence in threatened ex situ mammals. Cross-sectional studies can fall into Tier 1 if they measure microbiomes and outcomes in the same population, whereas larger or better-controlled longitudinal or experimental studies may be placed in Tier 2 or 3 when the linkage is less direct. This author-defined framework was created solely to map ecological and outcome directness across three levels: joint measurement in the same ex situ population, contextual evidence from the same conservation system, and external mechanistic support. It is complementary to, and not a substitute for, formal risk-of-bias assessment or certainty frameworks such as GRADE.

### 2.4. Exploratory Comparative Synthesis

**Figure 2:**
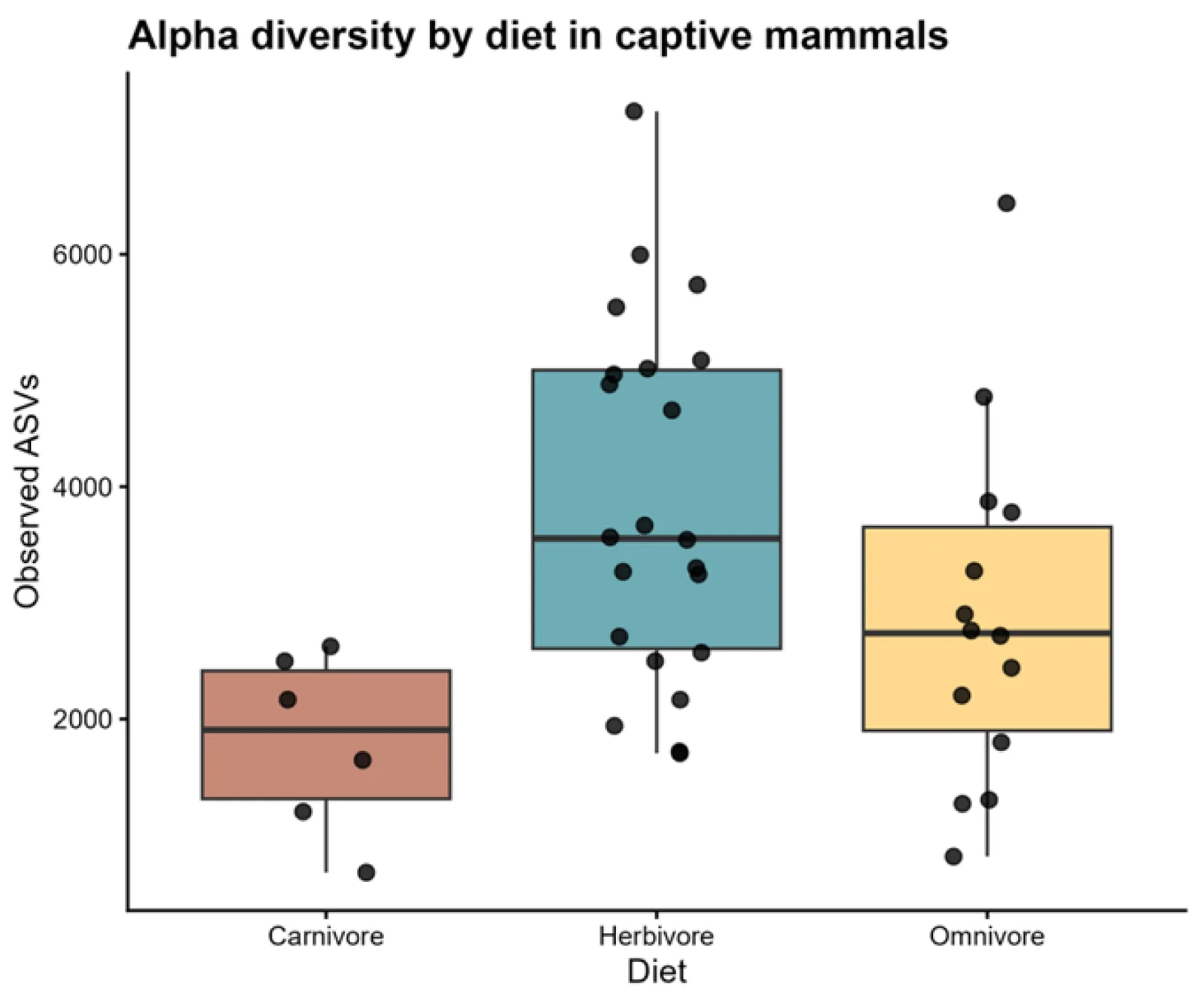
Species-level Observed ASV richness by dietary category in captive mammals. Each point represents one species-level mean. Colors correspond to dietary categories. Boxes represent the interquartile range, horizontal lines indicate medians, and whiskers extend to the most extreme values within 1.5 times the interquartile range. The figure illustrates an ecological pattern in the available dataset but does not control for phylogeny, gut morphology, sequencing depth, or study-specific processing.

**Figure 3:**
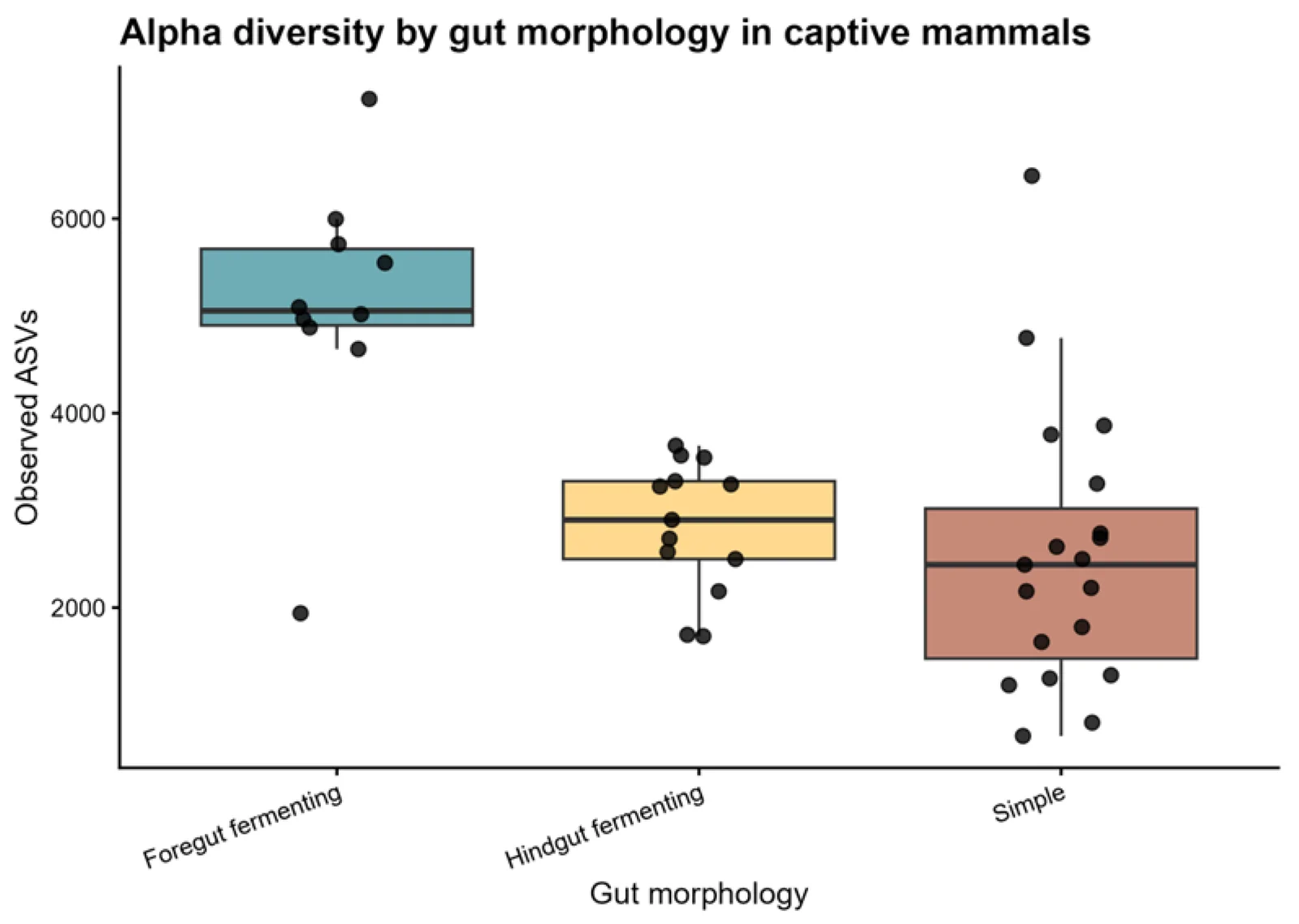
Species-level Observed ASV richness by digestive morphology in captive mammals. Each point represents one species-level mean. Colors correspond to digestive-morphology categories (foregut fermenters, hindgut fermenters, and mammals with a simple gut). Boxes represent the interquartile range, horizontal lines indicate medians, and whiskers extend to the most extreme values within 1.5 times the interquartile range. Foregut fermenters showed higher values in the source dataset, but diet, phylogeny, and digestive morphology are correlated and the pattern should be interpreted descriptively.

Published species-level mean values for observed amplicon sequence variants (Observed ASVs), observed species, or Chao1 were used only when the metric and management condition were identifiable. For richness across captive mammals, we used the captive subset reported by de Jonge et al. [44]. The source study included 70 fecal samples from 54 mammalian species overall, whereas the captive subset comprised 54 samples representing 42 species. Accordingly, the exploratory summaries in Table 2 and Figure 2 and Figure 3 are based on 42 captive species. The source study applied a common 16S rRNA analytical pipeline and assessed sequencing sufficiency using rarefaction curves; however, sequencing depth and the number of individuals represented per species were not standardized. These summaries should therefore be interpreted as approximate species-level descriptions rather than directly comparable reference values.

We summarized group means, standard deviations, medians, and distributions. These observations were not treated as confirmatory tests of diet or digestive anatomy because species are phylogenetically non-independent and the two host traits are strongly correlated. Similarly, published phylum-level relative abundances were used to describe the direction of within-host wild–captive differences. Relative-abundance data are compositional, and the underlying sequence data were not reprocessed through a common pipeline; therefore, the directional counts were interpreted as an evidence map rather than as estimates of a universal captivity effect. To support reproducibility, Supplementary Table S1 lists each primary study, the extracted values used in figures and narrative, and the exact table, figure, or page location from which those values were obtained. These steps were intended to provide a transparent descriptive synthesis and evidence map rather than a pooled quantitative effect estimate. In the 54-species dataset, richness estimates were based on amplicon sequence variants (ASVs), whereas other primary studies variously reported observed species or OTU-based richness. These metrics were not recomputed or pooled across pipelines; instead, they were used descriptively as published to illustrate relative differences in richness across dietary guilds and gut morphologies.

### 2.5. Figure Generation

**Figure 4:**
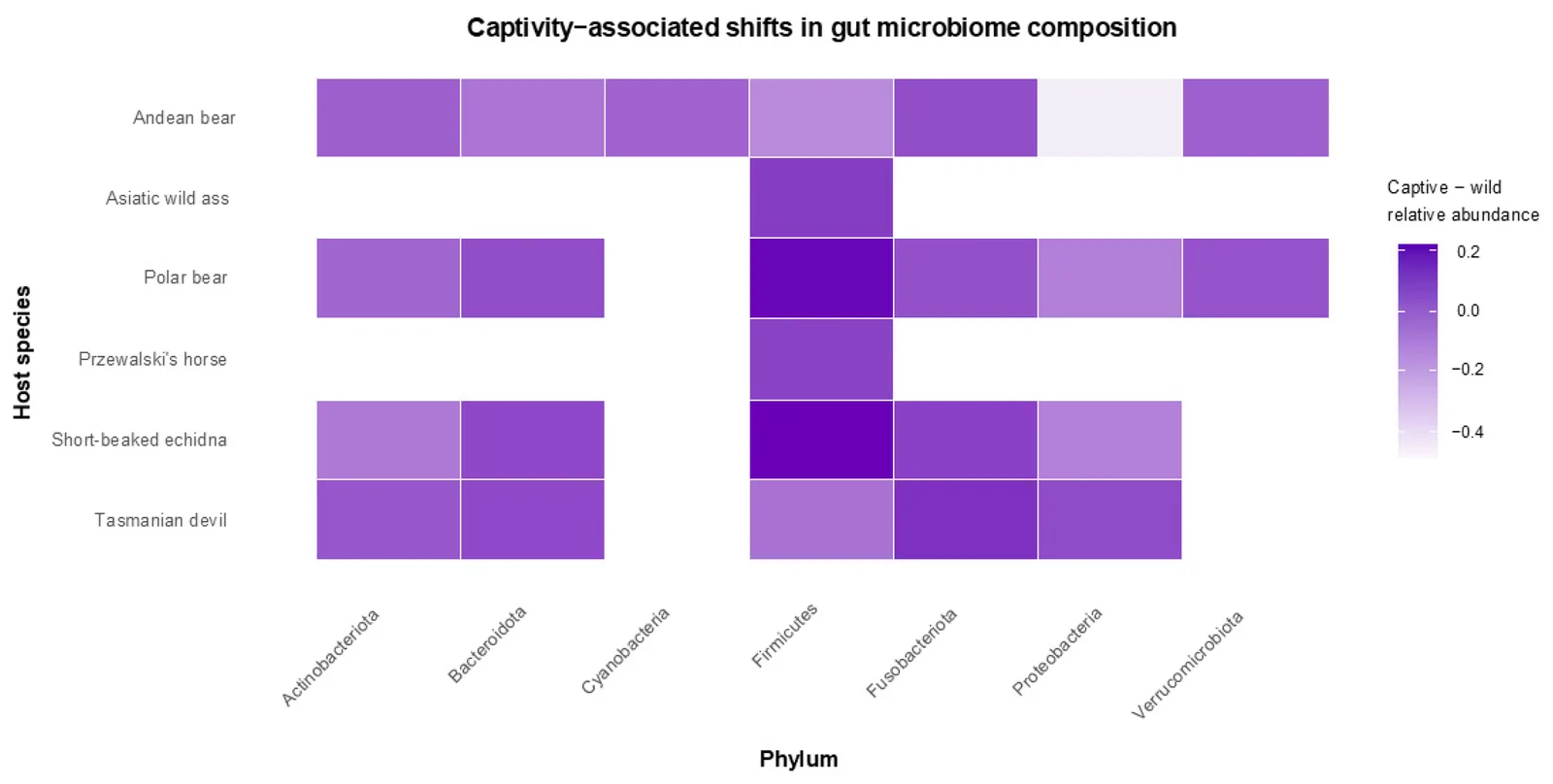
Direction and magnitude of published captive-minus-wild differences in major bacterial phyla. Values are based on reported mean relative abundances from within-host comparisons. Cell color indicates the captive-minus-wild difference in relative abundance; darker purple indicates a greater positive difference (higher relative abundance in captive animals), whereas lighter shades indicate a negative difference (lower relative abundance in captive animals). Blank cells indicate unavailable or non-comparable values, not absence of the phylum.

**Figure 5:**
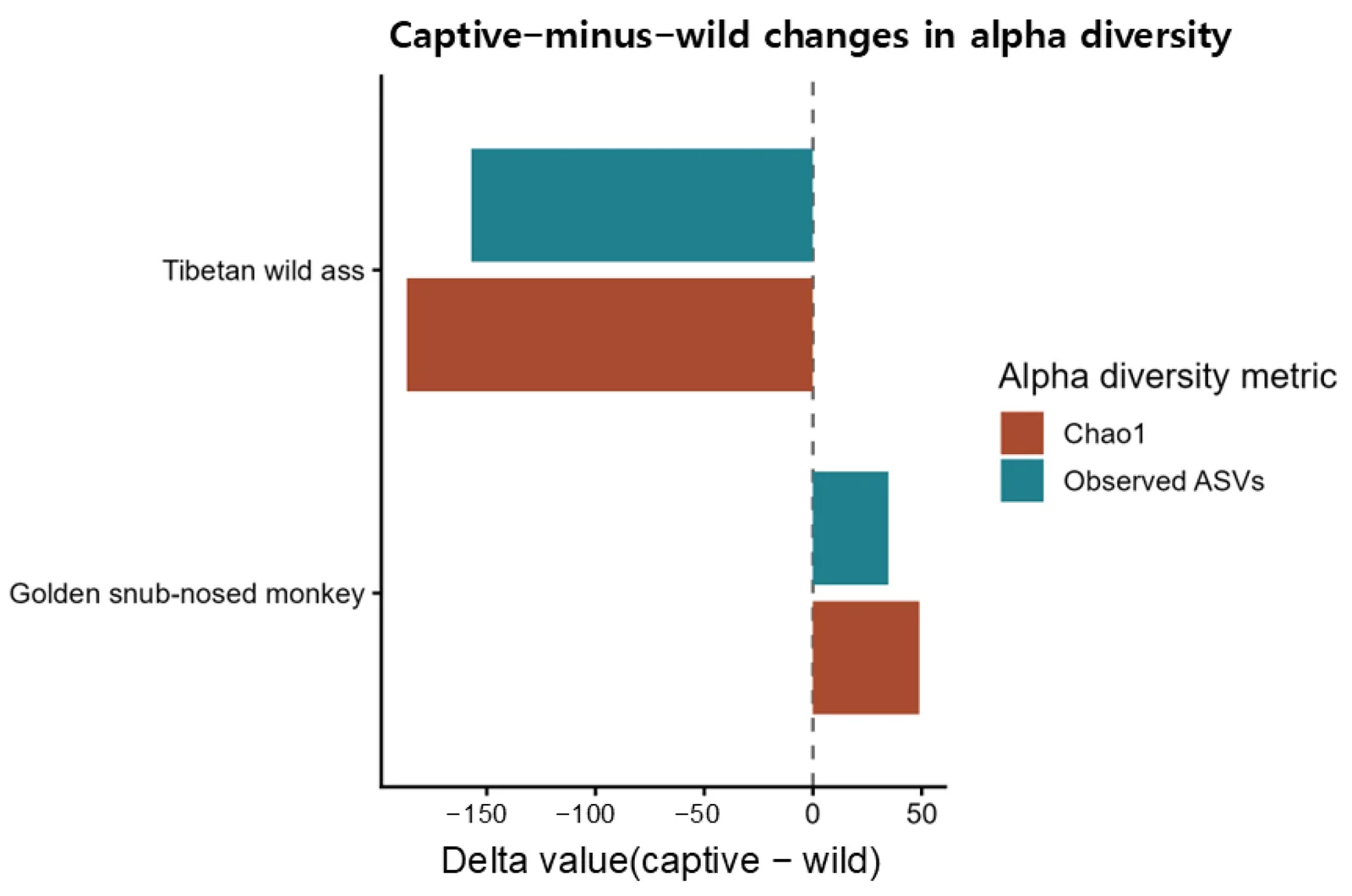
Published captive–wild differences in alpha diversity metrics for Tibetan wild asses and golden snub-nosed monkeys. Bars represent within-study captive-minus-wild differences in Chao1 richness (red) and observed ASVs (green). Negative values indicate lower richness in captive groups and positive values indicate higher richness. The figure shows opposite within-study responses and should not be used for between-study comparison of absolute values.

**Figure 6:**
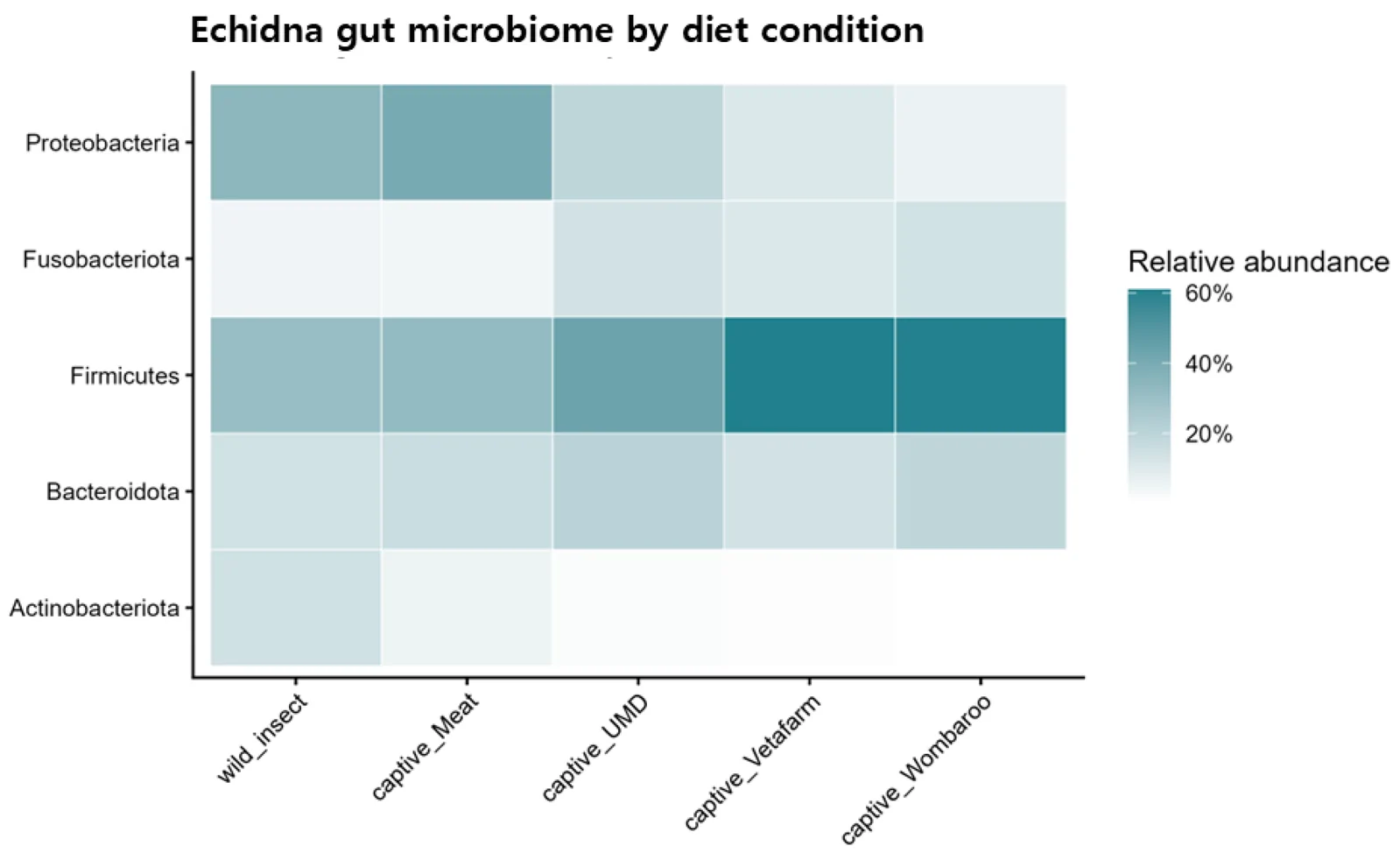
Major phylum-level relative abundances in short-beaked echidnas under wild insect-associated and four captive dietary conditions [32]. Rows represent major bacterial phyla, and columns represent dietary conditions. Cell color indicates relative abundance, with darker teal indicating higher relative abundance. White cells indicate unavailable values, as applicable. The heat map demonstrates diet-associated variation but does not identify an optimal or “healthy” diet without host outcome data. This figure illustrates patterns from a single study with small groups under four captive diets and one wild condition and should be interpreted as an example rather than a generalizable effect estimate.

**Figure 7:**
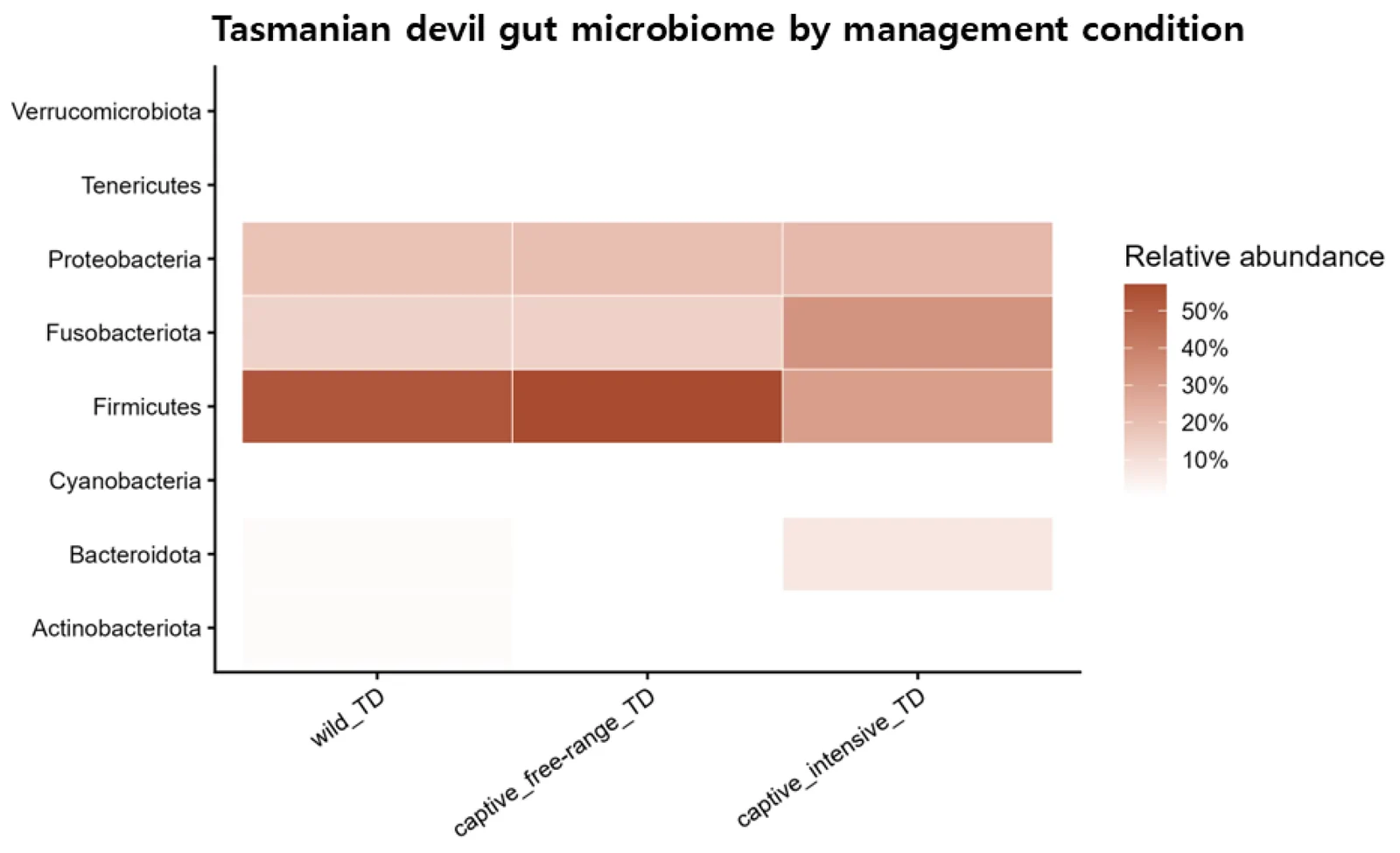
Major phylum-level relative abundances in wild, free-range captive, and intensively managed Tasmanian devils [24]. Rows represent major bacterial phyla, and columns represent management conditions. Cell color indicates relative abundance, with darker brown indicating higher relative abundance. White cells indicate unavailable values, as applicable. Free-range and wild profiles were more similar than the intensive-management profile, but small group sizes and correlated management variables limit causal inference. This figure presents results from a single Tasmanian devil study and is intended as an illustrative example of management-intensity differences, not a generalizable or causal effect size.

Figure 2, Figure 3, Figure 4, Figure 5, Figure 6 and Figure 7 were generated from numerical values extracted from the source text, tables, or supplementary files and compiled in R (version 4.5.1). No values were estimated visually from previously published plots. Figure 2 and Figure 3 display species-level Observed ASV values from the captive subset of de Jonge et al. [44], grouped by dietary guild and gut morphology, respectively. Figure 4 displays captive-minus-wild differences in reported mean phylum-level relative abundances for each host comparison. Figure 5 displays captive-minus-wild differences in reported alpha-diversity metrics for Tibetan wild asses and golden snub-nosed monkeys. Figure 6 and Figure 7 display reported mean phylum-level relative abundances across echidna diet conditions and Tasmanian devil management conditions, respectively. Data manipulation and plotting were performed using the R packages dplyr, tidyr, and ggplot2. The numerical values used in the exploratory synthesis and the source locations from which they were extracted are provided in Supplementary Table S1. The compiled dataset used to generate Figure 2, Figure 3, Figure 4, Figure 5, Figure 6 and Figure 7 is provided as Supplementary Table S2. All values were derived from the cited publications.

### 2.6. Cross-Study Limitations and Reproducibility

All cross-study comparisons are subject to variation in fecal collection, environmental exposure before collection, storage, DNA extraction, 16S rRNA target region, sequencing platform, rarefaction, denoising, taxonomic database, and statistical analysis [45,46,47]. Figures consequently visualize the range and direction of published observations. They do not establish a normal reference interval, a diagnosis of dysbiosis, or a transferable microbial biomarker.

The search strategy may under-represent articles not indexed in PubMed or highly ranked by Google Scholar, and the heterogeneous designs precluded use of a single validated risk-of-bias instrument. The tier framework was therefore used to distinguish direct ex situ evidence from contextual and mechanistic support, while study-specific limitations were reported in the text. The exploratory numerical synthesis should be viewed as a transparent evidence map rather than a formal meta-analysis. The documented two-author extraction and checking procedure, together with the source-level annotations in Supplementary Table S1, is intended to make the numerical synthesis reproducible for other investigators. Generative artificial intelligence was used to assist English-language editing, structural organization, and document formatting. All scientific interpretations, extracted values, citations, and final wording were reviewed by the authors, who take full responsibility for the manuscript.

## 3. Evidence Landscape and Baseline Variation

### 3.1. Host Ecology Precedes Captivity Effects

Gut microbiomes are products of host evolution as well as contemporary environment. Across mammals, host phylogeny, trophic strategy, gut anatomy, and the biochemical complexity of the diet contribute to community structure [48,49,50]. Herbivores generally require microbial fermentation of structural carbohydrates, whereas strict carnivores consume more digestible protein and fat and often have shorter, simpler gastrointestinal tracts. These differences influence substrate availability, retention time, redox conditions, and the ecological niches available to microorganisms. Any interpretation of an ex situ microbiome must therefore begin with host-appropriate expectations rather than with a single cross-species definition of diversity or health.

In the exploratory dataset of captive mammals, richness was highest among herbivorous species and lowest among carnivorous species. Mean Observed ASVs were 3864.5 in herbivores, 2882.7 in omnivores, and 1803.6 in carnivores. The same ordering was observed for Chao1. Foregut fermenters also showed higher species-level richness than hindgut fermenters and simple-gut species (Table 2; Figure 2 and Figure 3). These patterns are biologically plausible and consistent with the requirement for diverse fermentative functions in fiber-rich diets [44,50]. They should not, however, be interpreted as evidence that high richness is intrinsically healthier. Richness can be influenced by sequencing depth and bioinformatic decisions, and low richness may be a normal feature of a host adapted to a nutrient-dense carnivorous diet.

The apparent foregut-hindgut-simple gut pattern also illustrates the problem of correlated predictors. Most foregut fermenters in the dataset were herbivores, whereas simple-gut species included many carnivores and omnivores. In addition, related species share both digestive traits and microbiome features. A robust comparative analysis would require a phylogenetically informed multivariable model using consistently processed sequence data. For the present review, the distribution is best used as a reminder that host ecology defines the baseline against which management-associated change should be evaluated.

### 3.2. Why a Universal Captive Microbiome Is Unlikely

At broad taxonomic levels, many mammals are dominated by Firmicutes and Bacteroidota, but the relative abundance of these phyla varies with diet, gastrointestinal physiology, age, season, and analytical method. Captivity-related studies have reported changes in these and other phyla, including Actinobacteriota, Proteobacteria, Fusobacteriota, and Verrucomicrobiota [18,19,20,21,22,23,24,25,26,28,29,30,31,32,33,34,51]. The direction is not universal. In the limited host-level comparisons assembled here, Actinobacteriota was lower in all four captive comparisons in which it was reported, whereas Fusobacteriota was higher in four of four. Bacteroidota and Firmicutes changed in both directions, and Proteobacteria was usually but not invariably lower in captive groups. The study-level evidence map in Figure 4 shows that these shifts vary across hosts, whereas Table 3 condenses the same comparisons into directional counts for each phylum.

This directional concordance is hypothesis-generating rather than diagnostic. Four host-level comparisons are insufficient to define a general biomarker, and phylum-level aggregation can conceal opposite responses among constituent families or genera. Because relative-abundance data are compositional, an apparent increase in one group necessarily alters the proportions of others even if absolute microbial loads are unchanged. In addition, host identity is inseparable from study methodology because most species have been examined in a single facility or research project. Together, these limitations argue against using a Firmicutes:Bacteroidota ratio, Proteobacteria abundance, or any other broad taxonomic measure as a universal indicator of captivity-associated dysbiosis.

A more defensible interpretation is that captivity frequently reorganizes microbial community composition, while the resulting state is host- and context-specific. The conservation question is therefore not whether a captive microbiome is statistically different from a wild one, but whether the difference is reproducible within a management system and associated with meaningful outcomes such as digestion, inflammation, behavior, reproductive performance, or post-release survival.

## 4. Captivity as a Multilevel Ecological Filter

### 4.1. Transition from the Wild

Wild–captive transitions alter multiple ecological dimensions at the same time. Natural diets may be replaced by formulated feeds or restricted ingredient lists; foraging time and ranging behavior decline; exposure to soil, water, prey, vegetation, and wild conspecifics changes; and veterinary interventions become more frequent. Such transitions can remove microbial sources while creating new routes of acquisition from enclosures, food preparation areas, keepers, domestic animals, and other captive species. The resulting microbiome reflects a new metacommunity rather than a simple loss of wild taxa [18,21,22,34].

Alpha-diversity responses illustrate this complexity. Captive Tibetan wild asses showed lower Observed ASVs and Chao1 than wild animals, whereas captive golden snub-nosed monkeys showed higher observed richness and Chao1 than wild groups [26,52]. The captive-minus-wild values are shown in Figure 5. These studies used different sequencing and analytical pipelines, so their absolute values cannot be compared with one another. More importantly, opposite directions can both be biologically plausible. A restricted diet and reduced environmental exposure may decrease richness, whereas mixing of diets, environmental sources, or individuals from different origins may increase it. Alpha diversity alone therefore does not identify whether the change is adaptive, neutral, or harmful.

Beta-diversity and taxonomic composition more consistently distinguish captive from wild populations, but they also do not reveal mechanism. For example, Andean bears, polar bears, equids, echidnas, wombats, platypuses, and subterranean rodents each displayed captivity-associated community differences [19,23,25,26,27,28,32,51]. In several cases, diet was identified as an important correlate; in others, landscape, facility, health, or management history were also influential. Captivity is consequently better represented as a suite of interacting exposures than as a binary variable.

### 4.2. Diet as a Dominant but Context-Dependent Driver

Diet determines which substrates reach the distal gut and is among the most actionable drivers of microbiome composition in ex situ management. Natural diets can promote retention of native microbial features in captive rodents [48], whereas formulated diets may alter the relative representation of fiber-degrading, protein-fermenting, bile-tolerant, or short-chain-fatty-acid-producing organisms. Yet “natural” is not a single nutritional category. Seasonal wild diets may include hundreds of plant or prey items and substantial incidental soil or chitin, while a captive diet can be nutritionally complete but ecologically simplified. Conversely, a poorly characterized wild sample may reflect temporary food scarcity, anthropogenic feeding, or disease.

Short-beaked echidnas provide a clear example of diet-associated variation under managed conditions [32]. Wild animals classified under an insect-associated condition had relatively high Proteobacteria and Actinobacteriota. Captive meat, updated meat diet, Vetafarm, and Wombaroo conditions differed substantially, with Firmicutes exceeding 60% in the latter two diets and Actinobacteriota becoming uncommon (Figure 6). The comparisons indicate that feeding system can restructure the community, but they do not establish that one commercial or meat-based diet is superior. Functional measures, fecal quality, body condition, clinical outcomes, and nutrient digestibility would be required to determine management significance.

Dietary interpretation should also consider transition time. Microbiomes may respond within days to a new diet, whereas host physiology and stable microbial assembly can take longer. Abrupt dietary changes can therefore produce transient shifts that would be missed or misclassified in cross-sectional studies. For conservation breeding, repeated sampling before and after a planned diet change is more informative than comparison with a single wild reference sample. The same design can assess whether increased browse diversity, whole prey, fermentable fiber, or seasonal diet rotation produces a reproducible microbial and physiological response.

### 4.3. Enclosure, Management Intensity, and Environmental Microbial Exposure

Captive environments differ in enclosure size, substrate, vegetation, climate exposure, social structure, cleaning intensity, and opportunities for natural behavior. These factors can influence microbial acquisition directly and indirectly through activity, stress, diet selection, and contact networks. Enrichment with natural elements can modify compositional variation but may not fully prevent divergence from wild microbiomes [22]. This observation is important: a visually natural enclosure does not necessarily recreate the microbial exposures, movement patterns, or dietary heterogeneity of a wild habitat.

Tasmanian devils illustrate the effect of management intensity [24]. Wild and free-range captive groups had broadly similar phylum-level profiles dominated by Firmicutes, whereas intensively managed animals had lower Firmicutes and higher Fusobacteriota and Bacteroidota (Figure 7). The sample sizes for free-range and intensive groups were small, and diet and facility were intertwined with management condition. Nevertheless, the study shows why a binary captive-versus-wild classification can be misleading. Free-range conservation facilities, intensive breeding units, rescue hospitals, and zoo exhibits represent distinct ecological contexts.

Facility effects have also been observed in eastern black rhinoceroses and cheetahs [6,30]. Ex situ cheetahs managed in Namibia had higher bacterial diversity and distinct communities compared with animals managed in the United States, while retaining substantial overlap in abundant sequences [30]. Such findings can reflect diet, climate, substrate, antibiotic history, genetic structure, or sampling conditions. Multi-facility studies should therefore record management metadata at a resolution that permits these explanations to be separated. At minimum, this includes ingredient-level diet, feeding schedule, enclosure substrate, outdoor access, social grouping, cleaning agents, medication for the preceding months, body condition, gastrointestinal signs, reproductive status, and season.

### 4.4. A Multilevel Ecological-Filter Model

The evidence supports a multilevel ecological-filter model. The first filter is removal from the wild, which changes environmental sources, diet breadth, movement, and contact networks. The second filter consists of facility-level conditions such as climate, enclosure, substrate, and biosecurity. The third includes individual management events such as diet change, pairing, transport, antibiotic treatment, disease, pregnancy, and preparation for release. Host phylogeny and digestive physiology constrain responses at every level.

This model has two practical consequences. First, “wild-like” should be treated as a management hypothesis rather than an automatic target. A wild microbiome may include pathogens, reflect malnutrition, or be adapted to ecological pressures absent in captivity. Second, repeated within-individual measurements around defined management events are more interpretable than one-time comparisons among unrelated wild and captive animals. The management value of the microbiome lies in detecting reproducible change linked to outcomes, not in maximizing similarity to a single wild profile.

## 5. Microbiome Associations with Behavior, Stress, and Welfare

### 5.1. Biological Plausibility and Limits of Inference

Animal welfare is multidimensional and cannot be inferred from a single biological measure. Abnormal repetitive or stereotypic behaviors can indicate a history of frustration, restricted behavioral opportunities, or altered motivational systems, although their current welfare meaning depends on context and individual history [53,54,55]. Physiological stress is similarly complex: glucocorticoid concentrations vary with circadian and seasonal rhythms, metabolism, reproductive state, activity, and both negative and positive arousal. A fecal microbiome measure would therefore be valuable only as one component of an integrated assessment.

Microbiota can plausibly interact with stress and behavior through microbial metabolites, immune signaling, intestinal permeability, vagal pathways, and regulation of the hypothalamic–pituitary–adrenal axis [8,9,15,56,57,58]. Experimental studies demonstrate that stress can alter microbial communities and that microbiota transfer can modify behavioral phenotypes in recipient animals [15,16,59,60,61]. These mechanisms establish plausibility but do not prove that microbiome variation causes stereotypic behavior in an ex situ mammal. Diet, age, enclosure, season, and stress may independently influence both the microbiome and behavior, and the causal direction may be bidirectional.

### 5.2. Direct Evidence from Ex Situ Mammals

The most direct evidence currently comes from sun bears and captive macaques. In sun bears, behavioral observations and fecal 16S rRNA sequencing identified correlations between the occurrence of stereotypic behavior and the abundance of several bacterial taxa, including phylum- and genus-level features [5]. The study was valuable because it addressed an endangered species under ex situ management and used non-invasive samples. Its small population, seasonal structure, and correlational design mean that the reported taxa should be regarded as preliminary, hypothesis-generating candidates for replication rather than validated welfare biomarkers.

In rhesus macaques, animals with high levels of motor stereotypy differed in gut microbial beta diversity and immune-related gene expression from controls [62]. Lower relative abundance of the short-chain-fatty-acid-associated genus Phascolarctobacterium was reported in the stereotypy group. The combined microbiome and transcriptomic approach strengthens biological interpretation, but it remains possible that diet, activity, social history, or other individual characteristics contributed to both microbial and behavioral differences. Intervention studies are required to distinguish cause, consequence, and shared environmental drivers.

Tier 3 evidence supports measurement of stress endocrinology alongside microbiomes. In black howler monkeys living in disturbed forest fragments, lower food availability, fecal glucocorticoid metabolites, and gut bacterial community structure were interrelated [63]. Fish and bird studies have also reported glucocorticoid-associated microbial signatures [64,65]. These studies are not direct evidence for zoo mammals, but they show that environmentally induced endocrine variation can covary with gut microbial ecology. For ex situ applications, fecal glucocorticoid metabolites should therefore be analyzed together with diet, activity, reproductive state, and repeated microbiome samples rather than used as an isolated validation measure.

### 5.3. Implications for Welfare Monitoring

The microbiome is best positioned as an adjunctive welfare measure [66]. A useful monitoring program would combine a validated ethogram, individual-level frequency and context of abnormal repetitive behavior, fecal glucocorticoid metabolites, fecal quality, body condition, relevant clinical data, and repeated microbiome profiles. Sampling should span baseline, intervention, and follow-up periods. Potential interventions include environmental enrichment, altered feeding schedule, diet diversification, social reorganization, treatment of chronic gastrointestinal disease, or transfer to a more complex enclosure.

The desired endpoint is not a predetermined taxonomic composition. Instead, evidence of management benefit would include improved behavioral diversity or reduced harmful repetitive behavior, an appropriate endocrine response, improved gastrointestinal or metabolic health, and a reproducible microbiome change that is temporally associated with the intervention. Replication across individuals and, where possible, facilities would be needed before any feature could be used prospectively. Until such evidence exists, terms such as “microbial welfare biomarker” should be reserved for validated prediction, whereas “candidate monitoring feature” is more accurate.

## 6. Microbiome Associations with Reproductive Physiology and Performance

### 6.1. Reproductive State, Hormones, and Gut Microbiome

Reproduction is a central performance outcome for conservation breeding populations. It is also a major physiological transition involving changes in sex steroids, energy balance, immune regulation, appetite, social behavior, and stress responsiveness. Each of these factors can alter the gut microbiome, while microbial metabolism may in turn influence energy extraction, inflammatory tone, and enterohepatic metabolism of hormones [7,10,17,42,43]. The relationship should therefore be conceptualized as a bidirectional network rather than a one-way gut–reproduction axis.

Eastern black rhinoceroses provide the strongest direct ex situ example [6]. Fecal bacterial communities differed among individuals, institutions, breeding-success categories, and ovarian-cycle phases. Pregnancy and the post-parturition period were associated with community changes, and several low-abundance taxa—including Aerococcaceae, Atopostipes, Carnobacteriaceae, and Solobacterium—were associated with breeding success, reproductive state, or higher fecal progestagen metabolites. Many genera also correlated with progestagen or glucocorticoid metabolites. The study demonstrates the value of integrating microbiomes with non-invasive endocrinology, but it does not establish that the identified taxa improve fertility. Institution and individual identity were substantial sources of variation, and the direction of the microbiome–hormone relationship remains uncertain.

Black-footed ferrets provide evidence for sex-specific seasonal dynamics [67]. Male and female gut bacterial communities were relatively similar during the non-breeding period but diverged during the breeding season. This finding is consistent with sex-specific reproductive physiology, behavior, and energy expenditure. It also warns against defining a static species reference microbiome without accounting for sex and season. In seasonally breeding species, a change that appears abnormal relative to an annual average may be a normal reproductive transition.

### 6.2. Multigenerational Captive Adaptation and Fitness

A particularly important advance is the multigenerational study of the endangered Pacific pocket mouse [68]. During establishment of a conservation breeding and reintroduction program, gut microbiome composition shifted gradually and stabilized into a captivity-associated state after approximately two to three generations. These transitions paralleled changes in body mass and reproductive performance, and microbial taxa associated with successful reproduction were identified. Unlike a single cross-sectional comparison, this design captured temporal dynamics across five generations and showed that microbial adaptation to captivity may not be immediate.

The Pacific pocket mouse findings have major implications for ex situ management. First, animals born in captivity cannot be assumed to have the same microbial trajectory as wild founders. Second, generation number may confound comparisons among facilities or release cohorts. Third, microbiome changes can coincide with apparent fitness changes without demonstrating causation, because genetic adaptation, maternal transmission, diet, facility maturation, and management experience may all change at the same time. Future programs should therefore archive fecal samples and detailed metadata from founders and descendants, allowing later analyses of host genetics, microbial transmission, and reproductive performance.

### 6.3. Stress, Pair Stability, and Reproductive Context

Reproductive success is also shaped by social compatibility and stress. In captive Vancouver Island marmots, established pairs and successful breeding pairs had lower fecal glucocorticoid metabolite concentrations than newly formed or unsuccessful pairs in relevant analyses [69]. That study did not jointly measure the gut microbiome and therefore constitutes contextual rather than direct evidence. A separate study of Vancouver Island marmots found that captivity conditions influenced gut microbial communities and traits relevant to hibernation and reintroduction preparation [20]. Together, these studies identify a conservation system in which integrated longitudinal measurement of pair behavior, fecal glucocorticoids, reproductive hormones, microbiomes, hibernation, and breeding outcomes would be especially informative.

Free-ranging primates further demonstrate that reproductive states can coincide with microbiome change. Female Tibetan macaques, Phayre’s leaf monkeys, and capuchin monkeys showed community variation across cycling, pregnancy, lactation, or seasonal reproductive states [70,71,72]. Experimental work in Brandt’s voles linked photoperiod, seasonal breeding, and gut microbiota [73]. These studies strengthen biological plausibility but cannot be transferred directly to ex situ endangered species. Their principal contribution is to identify design variables—reproductive stage, photoperiod, activity budget, diet, and season—that must be measured in conservation breeding studies.

### 6.4. What Can Currently Be Monitored?

No taxon can currently be recommended as a universal microbial marker of fertility. The taxa associated with rhinoceros breeding, ferret breeding season, or pocket-mouse reproductive performance were identified in different hosts with different diets, reproductive systems, and analytical pipelines. Even within a species, facility and individual effects can exceed the effect of reproductive category. A practical program should therefore focus on within-species, within-facility trajectories.

For females, useful sampling points may include pre-breeding baseline, follicular or estrous phase, luteal phase, early and late pregnancy, parturition, lactation, pregnancy loss, and return to cyclicity. For males, sampling may span non-breeding and breeding seasons, changes in testosterone or gonadal activity, semen collection, pairing, and confirmed paternity. Microbiome data should be integrated with fecal progestagen, androgen and glucocorticoid metabolites, body condition, diet intake, reproductive behavior, ultrasonography or semen quality where feasible, antimicrobial exposure, and reproductive outcome. Repeated samples are essential because a single fecal sample cannot distinguish a stable state from short-term fluctuation. The evidence tiers for interpreting microbiome associations with welfare and reproductive outcomes relevant to ex situ mammal conservation are summarized in Table 4.

## 7. From Association to Application

### 7.1. An Integrated Monitoring Framework

**Figure 8:**
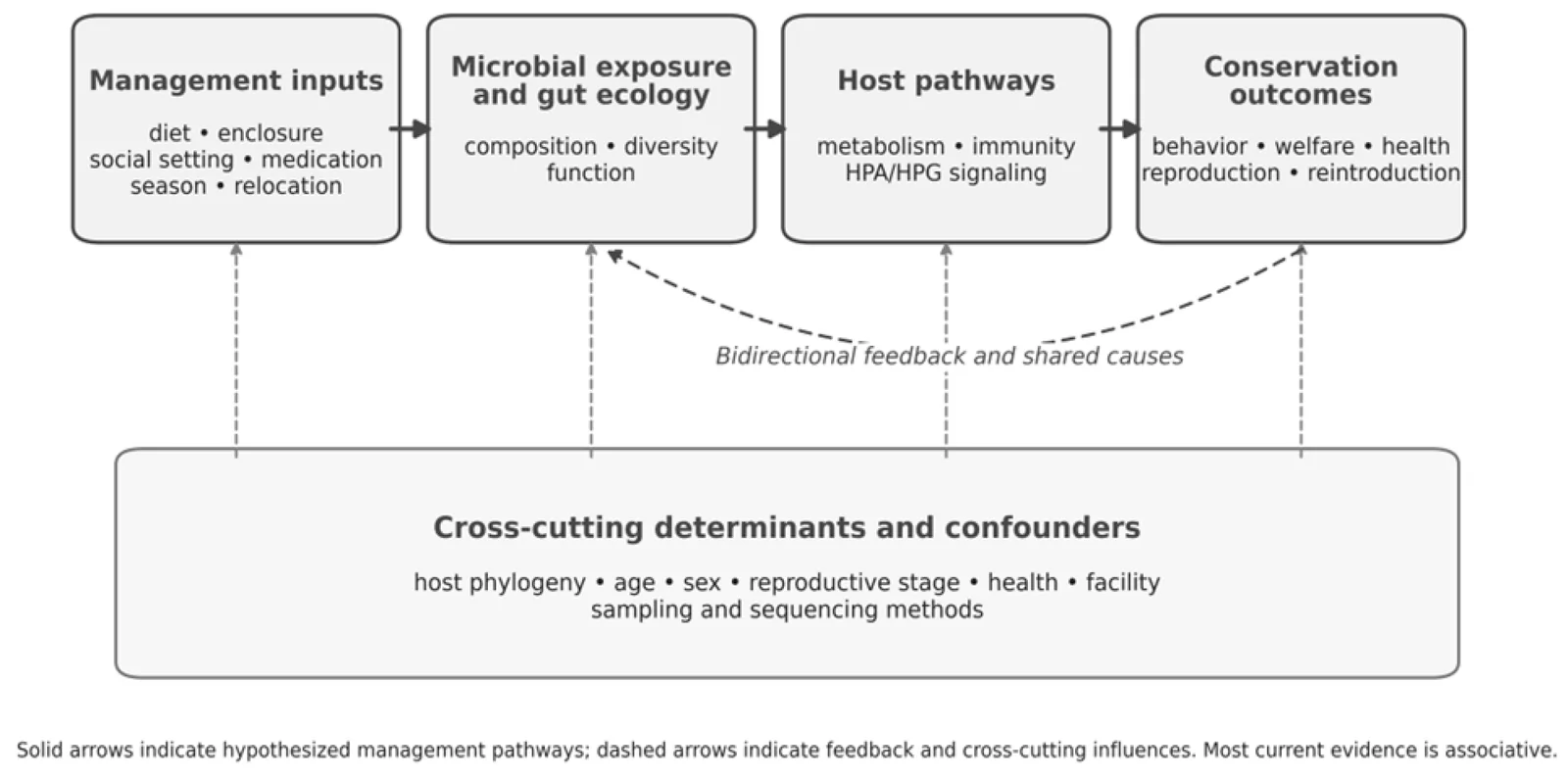
Integrated framework for microbiome-informed management of ex situ mammals. The upper pathway illustrates the proposed sequence from management inputs (e.g., diet, enclosure, social setting, medication, season, and relocation), through microbial exposure and gut ecology and host pathways, to conservation outcomes. The lower box summarizes cross-cutting determinants and potential confounders, including host phylogeny, age, sex, reproductive stage, health status, facility effects, and sampling and sequencing methods. Solid arrows represent hypothesized management pathways; dashed arrows represent feedback and cross-cutting influences. Current evidence is primarily associative, and host or methodological factors can affect every stage of the framework.

The microbiome should be incorporated into conservation management as a context-dependent physiological data stream, not as a stand-alone diagnostic. Figure 8 presents an integrated framework. Management inputs—including diet, enclosure, social setting, medication, season, and relocation—alter microbial exposure and gut ecology. Microbial composition and function may interact with metabolism, immunity, and the hypothalamic–pituitary–adrenal and hypothalamic–pituitary–gonadal axes. These pathways can be associated with behavior, welfare, health, reproductive performance, and reintroduction readiness. At every stage, host phylogeny, age, sex, reproductive state, facility, health, and laboratory methodology can create or modify associations.

A minimum longitudinal protocol could include three baseline samples, repeated sampling during a defined intervention or physiological transition, and follow-up after a biologically justified interval. For microbiome monitoring, fecal samples should be collected and processed using consistent, well-documented procedures. Sampling method, subsampling location within the fecal bolus, environmental exposure after defecation (time, temperature, substrate), storage buffer, freezing delay, and homogenization can all materially affect the observed community composition. As a minimum, fresh feces should be sampled as soon as feasible after defecation, avoiding obvious external contamination, with the internal portion of the bolus homogenized thoroughly before aliquoting into sterile tubes. The chosen storage buffer, storage temperature, and maximum delay to freezing or stabilization should be standardized within a study and reported explicitly. Aliquots should be stored in a way that minimizes freeze–thaw cycles [30,45]. Metadata should be collected at the individual level. For multi-institution studies, common standard operating procedures, extraction controls, negative controls, mock communities, and balanced sequencing batches are essential.

The analytical endpoint should be selected before sampling. If the question concerns monitoring within a population, consistent 16S rRNA sequencing may be sufficient to detect compositional trajectories. If the goal is mechanism or intervention, shotgun metagenomics, microbial load, metabolomics, short-chain fatty acids, bile acids, inflammatory markers, or culture-based functional testing may be required. Functional inference from 16S profiles alone should be interpreted cautiously, particularly in non-model hosts with poorly represented microbial genomes [40].

### 7.2. Candidate Applications

Four applications are realistic in the near term. The first is evaluation of diet change, particularly when fecal quality, body condition, gastrointestinal disease, or poor reproductive performance suggests a nutritional problem. The second is integrated welfare assessment around enclosure renovation, enrichment, social regrouping, or transport. The third is longitudinal reproductive monitoring, especially in seasonally breeding or repeatedly non-breeding individuals. The fourth is preparation for reintroduction, where microbiomes can be tracked during exposure to natural foods, outdoor substrates, reduced human contact, and post-release conditions.

Intervention with probiotics, prebiotics, or fecal microbiota transfer is more speculative. Candidate taxa associated with breeding or health may be markers rather than causal agents, and organisms beneficial in one host may be ineffective or pathogenic in another. Wildlife-specific products also raise biosafety, antimicrobial resistance, regulatory, and ecological-release concerns [36,38,39]. Manipulation should therefore follow mechanistic evidence, genome-level safety assessment, and controlled trials with clinically meaningful endpoints. Dietary and environmental approaches that support endogenous microbial functions are likely to be more acceptable initial interventions than direct microbial transplantation.

### 7.3. Reintroduction and Microbial Preparedness

Reintroduction exposes animals to novel foods, pathogens, competitors, climate, and energetic demands. A microbiome shaped by generations in captivity may influence adaptation, but similarity to wild conspecifics should not be the sole criterion of readiness. A useful assessment would test whether animals can transition toward a functionally appropriate community while maintaining body mass, fecal quality, immune competence, behavioral flexibility, and endocrine stability. Studies of obligate hibernators and multigenerational captive populations show that facility and generation can shape microbial trajectories relevant to release planning [20,68].

Pre-release programs should collect samples before natural-diet exposure, during acclimatization, immediately before release, and after release where non-invasive monitoring is possible. Released and non-released controls, or multiple release cohorts, can help distinguish seasonal change from the effect of preparation. Linking microbiome trajectories with survival, movement, foraging, disease, and reproduction would provide the outcome validation currently missing from most conservation microbiome studies.

## 8. Methodological Limitations and Research Priorities

### 8.1. Standardization Without Oversimplification

Technical variation remains a central barrier to synthesis. Sample age and environmental exposure can change fecal communities; extraction kits differ in lysis efficiency; primers capture different bacterial groups; and OTU versus ASV approaches alter diversity estimates [30,45,46,47]. Standardization should therefore focus on transparent reporting and compatible protocols within collaborative studies rather than assuming that all historic data can be directly pooled. Raw sequence data, metadata dictionaries, and analysis code should be deposited whenever ethical and legal conditions permit.

Given the small number of Tier 1 studies and their observational, taxon-specific designs, the following protocol should be viewed as a set of pragmatic recommendations for exploratory monitoring and method standardization, not as a prescriptive or fully validated diagnostic scheme.

Controls are especially important in low-biomass or field-collected samples. Negative extraction and PCR controls, positive mock-community controls, sample randomization, and batch information should be reported. For fecal samples, time since defecation, ambient temperature, rainfall, and substrate contamination may be relevant. Studies should avoid presenting predicted function from taxonomic data as equivalent to measured metabolic activity.

### 8.2. Study Design for Small Endangered Populations

Large sample sizes are rarely possible in threatened species, but strong inference can still be achieved through repeated measures, planned management events, and hierarchical analysis. Each individual can serve as its own reference across diet change, pairing, breeding season, pregnancy, treatment, or relocation. Multiple baseline samples are needed to estimate natural temporal variation. Mixed-effects models can separate within-individual change from differences among individuals and institutions.

Where several facilities manage the same species, prospective harmonization is particularly valuable. Balanced recruitment across sex, age, reproductive status, and institution can reduce confounding. Facility should be treated as a biological exposure, not merely as a nuisance variable, because it captures diet, climate, substrate, microbial sources, and management history. Host relatedness and pedigree can also be incorporated, especially in intensively managed breeding populations.

### 8.3. Move from Taxonomy to Function and Prediction

Most wildlife studies remain based on 16S rRNA amplicon profiles. This approach is appropriate for community description but often cannot identify strain-level function, antimicrobial resistance, viruses, fungi, or metabolic pathways. Shotgun metagenomics and metatranscriptomics can provide more direct functional information, while targeted metabolomics can test whether microbial products such as short-chain fatty acids, bile acids, indoles, or steroid metabolites covary with host outcomes [40]. The added cost is justified when the study aims to establish mechanism or intervention rather than surveillance alone. Where studies used different 16S processing schemes (for example, OTUs versus ASVs), richness metrics were interpreted within studies rather than as directly comparable absolute values across pipelines.

Prediction should be evaluated prospectively. A candidate signature associated retrospectively with breeding success must be tested in a new breeding season or facility before it is considered a biomarker. Performance should be reported using sensitivity, specificity, calibration, and predictive value, with clear separation of training and validation data. Because population sizes are small, parsimonious models based on ecological functions or within-individual change may generalize better than high-dimensional taxonomic classifiers.

### 8.4. Causality, Welfare, and Ethical Safeguards

Current evidence rarely distinguishes whether microbiome change causes an outcome, results from it, or shares a common driver. Causal inference can be strengthened through temporal ordering, repeated measurement, mediation analysis, and controlled intervention. In wildlife, direct microbiome manipulation may not be necessary for every question; management interventions such as diet modification or environmental enrichment can serve as ethically appropriate perturbations if the microbiome and host outcomes are measured before and after treatment.

Welfare safeguards must remain primary. Sampling should be non-invasive whenever possible and should not increase handling solely to obtain microbiome data. Microbial interventions require pathogen screening, antimicrobial-resistance assessment, and consideration of effects on recipients, keepers, conspecifics, and ecosystems after release. The conservation value of information must justify the burden and risk of any intervention. Across this literature, most reported microbiome–outcome links remain associative and should be treated as design signals and candidate monitoring features until they are replicated, temporally ordered, and supported by mechanistic or interventional evidence.

### 8.5. Priority Research Questions

- Which within-individual microbiome changes are reproducibly associated with improvements or deterioration in validated welfare outcomes?
- Do diet diversification, natural substrates, or reduced management intensity produce functional benefits rather than merely greater similarity to wild communities?
- Can microbiome trajectories predict pregnancy, pregnancy loss, successful parturition, offspring survival, or male reproductive performance after accounting for hormones, age, facility, and season?
- How are founder microbiomes transmitted and reorganized across generations in conservation breeding populations?
- Does pre-release microbial preparation improve post-release diet adaptation, health, survival, or reproduction?
- Which microbial functions, rather than taxonomic identities, are conserved across host species and management systems?

## 9. Conclusions

Captivity frequently changes the composition of mammalian fecal microbiomes, but the direction and biological meaning of change are not universal. Host phylogeny, diet, digestive anatomy, facility, season, reproductive status, and technical methodology all shape the observed community. The available evidence does not support a single captive dysbiosis signature or a transferable bacterial biomarker of welfare or fertility.

Direct associations with stereotypic behavior, reproductive hormones, breeding season, breeding success, and multigenerational fitness suggest that microbiome variation can be biologically relevant in selected conservation systems. However, these associations are based on small, largely observational studies and do not yet demonstrate that microbiome measures are consistently predictive, reproducible across populations, or sufficient on their own for management decisions. At present, its most realistic contribution is as an exploratory, non-invasive longitudinal measure integrated with behavior, endocrinology, clinical assessment, nutrition, and reproductive history, rather than as a validated welfare or fertility biomarker. The next phase of research should move from one-time taxonomic description toward standardized repeated measures, functional profiling, prospective prediction, and management interventions with explicit host outcomes. Used in this way, microbiome data can strengthen conservation physiology and evidence-based ex situ management without overstating causality.

## Figures and Tables

**Table 1:** This review in relation to recent microbiome reviews.

Review	Primary Focus	Host/System	What Is Missing for Ex Situ Mammals
Diaz and Reese 2021 [38]	Using gut microbiome to improve captive animal health	Captive animals broadly (zoo, lab, domestic)	No structured synthesis for threatened ex situ mammals, welfare, and breeding together
Dallas and Warne 2023 [39]	Microbiota in animal conservation	Conservation biology in general	Conceptual; does not map quantitative ex situ mammal evidence
Wang et al., 2025 [40]	Conservation metagenomics of threatened mammals	Threatened mammals (any setting)	Genomics-focused; not centered on welfare or breeding in managed populations
Cavalcante et al., 2025 [41]	Reproductive microbiomes and conservation	Reproductive-tract microbiomes	Reproductive tract, not gut; no cross-taxon view of gut–welfare–breeding links
This review	Gut microbiomes in ex situ-managed mammals	Ex situ mammals in zoos, breeding, and release programs	Integrates wild–captive shifts, within-captive management, welfare, stress, and reproductive performance

The present review is distinguished from prior syntheses by its explicit focus on ex situ-managed mammals and on links between gut microbiomes, welfare indicators, and conservation breeding outcomes.

**Table 2:** Exploratory species-level summary of gut microbial richness estimates in captive mammals.

Variable	Group	n	Mean (Observed ASVs)	SD	Median	Mean (Chao1)	SD	Median
Diet	Carnivore	6	1803.6	766.6	1907.2	2345.0	925.3	2522.5
Diet	Herbivore	22	3864.5	1541.3	3554.0	5117.0	1918.9	4820.8
Diet	Omnivore	14	2882.7	1503.5	2739.5	3777.6	2181.3	3603.5
Gut morphology	Foregut fermenter	10	5105.8	1340.9	5052.2	6649.0	1634.4	6812.6
Gut morphology	Hindgut fermenter	13	2835.6	674.7	2902.0	3860.7	927.5	4105.0
Gut morphology	Simple gut	19	2540.9	1435.5	2441.0	3307.9	2029.7	3149.0

Values are based on published species-level means from the captive subset of de Jonge et al. [44], comprising 54 fecal samples from 42 mammalian species. The diet (carnivore, omnivore, herbivore) and gut-morphology (foregut fermenter, hindgut fermenter, simple gut) group sizes therefore sum to 42, corresponding to the 42 captive species represented in that subset.

**Table 3:** Directional concordance of major phylum-level differences in available within-host wild–captive comparisons.

Phylum	Lower in Captive	Higher in Captive	Interpretive Caution
Actinobacteriota	4	0	Only four host-level comparisons
Bacteroidota	1	3	Direction varied among hosts
Cyanobacteria	1	0	Single comparison
Firmicutes	2	4	Direction varied; broad phylum
Fusobacteriota	0	4	Carnivore representation may influence pattern
Proteobacteria	3	1	Relative abundance is not equivalent to pathogen burden
Verrucomicrobiota	2	0	Only two comparisons

Directional counts derived from the host-level wild–captive comparisons shown in Figure 4.

**Table 4:** Evidence tiers for microbiome links with welfare and reproductive outcomes relevant to ex situ mammal conservation.

Domain	Host Species and Study Context	Variables Measured Together	Evidence Tier	Main Findings and Limitation
Stereotypic behavior	Sun bear (*Helarctos malayanus*), ex situ-managed population	Stereotypic behavior and fecal microbiome	Tier 1	Associations between bacterial taxa and pacing frequency were reported; the study involved a small managed population and did not include a causal intervention [5].
Motor stereotypy and immune status	Rhesus macaque (*Macaca mulatta*), captive population	Motor stereotypy, fecal microbiome, and transcriptome	Tier 1	Microbial community and immune-related differences were associated with stereotypy; residual cross-sectional confounding limits causal interpretation [62].
Stress ecology	Black howler monkey (*Alouatta pigra*), free-ranging populations in disturbed forest fragments	Food availability, fecal glucocorticoid metabolites, and fecal microbiome	Tier 3	Provides mechanistic support from free-ranging wildlife, but it does not represent an ex situ population [63].
Breeding success and ovarian state	Eastern black rhinoceros (*Diceros bicornis michaeli*), ex situ-managed population	Fecal microbiome, progestagen and glucocorticoid metabolites, ovarian state, and breeding outcome	Tier 1	Integrated associations were detected, but institution-level and individual effects were substantial and directionality remains unresolved [6].
Breeding season	Black-footed ferret (*Mustela nigripes*), ex situ conservation population	Sex, breeding season, and fecal microbiome	Tier 1	Sex-specific seasonal shifts were identified; reproductive performance was not experimentally manipulated [67].
Multigenerational fitness	Pacific pocket mouse (*Perognathus longimembris pacificus*), multigenerational conservation-breeding population	Five captive generations, fecal microbiome, body mass, and reproductive performance	Tier 1	Microbiome trajectories paralleled changes in fitness-related traits, but several captive conditions changed simultaneously [68].
Pair stress and breeding	Vancouver Island marmot (*Marmota vancouverensis*), captive breeding-for-release program	Fecal glucocorticoid metabolites and breeding outcomes; the microbiome was characterized in a separate study	Tier 2	The species is well suited for integrated follow-up, but current evidence is derived from separate datasets [20,69].
Reproductive states and seasonal breeding	Tibetan macaque (*Macaca thibetana*), free-ranging population; Phayre’s leaf monkey (*Trachypithecus phayrei crepusculus*), free-ranging population; White-faced capuchin (*Cebus capucinus imitator*), free-ranging population; Brandt’s vole (*Lasiopodomys brandtii*), experimentally housed population	Reproductive state or photoperiod, hormones or activity, and fecal microbiome	Tier 3	These studies support biological plausibility and identify key design variables, but their transferability to ex situ conservation populations remains uncertain [70,71,72,73].

Tier 1: the microbiome and the target outcome were measured in the same ex situ-managed population. Tier 2: contextual ex situ evidence was derived from partially overlapping or separate datasets. Tier 3: mechanistic support was derived from free-ranging wildlife, domestic species, or experimental models. The tiers describe the directness of outcome linkage in this review and should not be interpreted as rankings of methodological quality, risk of bias, or certainty of evidence.

## Data Availability

No new sequence data were generated. All numerical values used in the exploratory synthesis were obtained from the cited publications and are summarized in the tables and figures of this manuscript.

## Supplementary Materials

The following supporting information can be downloaded at: https://www.mdpi.com/article/10.3390/ani16162480/s1, Table S1: Literature search strategy, included studies, extracted numerical values, and source-level annotations; Table S2: Compiled numerical dataset used to generate Figure 2, Figure 3, Figure 4, Figure 5, Figure 6 and Figure 7.
